# Clinicopathological abnormalities and outcome of acute *Babesia canis* infections in 23 dogs treated with imidocarb dipropionate

**DOI:** 10.1016/j.crpvbd.2026.100395

**Published:** 2026-06-02

**Authors:** Clara M. Eisenecker, Andreas Moritz, Imke M. von Hohnhorst, Christina Strube, Elisabeth Müller, Ingo Schäfer

**Affiliations:** a Small Animal Clinic – Internal Medicine, Department of Veterinary Medicine, Justus-Liebig-University, Giessen, Germany; b Clinical Pathology and Clinical Laboratory Diagnostics, Department of Veterinary Medicine, Justus-Liebig-University, Giessen, Germany; c Institute for Parasitology, Centre for Infection Medicine, University of Veterinary Medicine Hannover, Hanover, Germany; d Laboklin GmbH & Co. KG, Bad Kissingen, Germany

**Keywords:** Antiprotozoal, Piroplasm, *Babesia*, Thrombocytopenia, Ticks

## Abstract

*Babesia canis* infections are considered an emerging tick-borne disease most often with unspecific clinical signs. Two injections of 6.6 mg/kg bodyweight (BW) imidocarb dipropionate (ID) in a timeframe of 14 days are recommended for treatment. *Babesia* antibody levels are protective against severe disease. This prospective study aimed to describe clinicopathological abnormalities and *Babesia* antibody levels in 23 dogs with acute *B. canis* infections, defined by positive PCR sequencing results revealing *B. canis* on EDTA-blood, and to follow these dogs from T0 (diagnosis, first ID) to T1 (laboratory control, second ID), and T2 (laboratory control). Complete blood counts, biochemistry, coagulation profiles, Coombs’ testing, and *Babesia* spp. antibody levels were determined. Thrombocytopenia, anemia, hyperbilirubinemia, and acute phase-responses were the most detected. ID dosage ranged from 1.7 to 7.0 mg/kg BW (median: 4.2 mg/kg BW). All PCR-results were negative at T1 and T2. At T1 (median 15 days after T0), laboratory abnormalities significantly improved but no further improvement was noted comparing T1 to T2 (median 32 days after T0). Initially serologically negative dogs built up *Babesia* antibody levels by T1, which mostly dropped by T2. All 23 dogs were PCR-negative at T1 besides the wide range in ID dosage. Laboratory abnormalities resolved quickly and all dogs had an uncomplicated course of disease. Dogs should be checked by PCR 14 days after the first imidocarb dipropionate injection. If the PCR result is negative and clinicopathological abnormalities have significantly improved, a second injection is not recommended to avoid a decrease in protective antibody levels.

## Highlights

• *Babesia canis* should not be ruled out in dogs without fever and anemia.
• Marked thrombocytopenia was present in all 23 dogs with *B. canis* infection.
• Decrease in *Babesia* antibody levels after second imidocarb dipropionate injection.
• PCR two weeks after the first imidocarb dipropionate injection is recommended.
• Identification of subclinical carriers is important to prevent the spread of the pathogen.

## Graphical abstract

**Figure:**
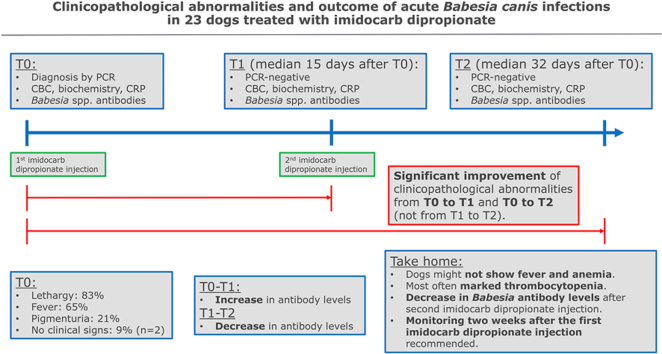

## 1 Introduction

Canine babesiosis can be caused by several *Babesia* species with different tick species as vectors, different geographical distribution and different pathogenicity (Leisewitz et al., 2023). The most important species of *Babesia* endemic in Europe include *Babesia canis*, *Babesia vogeli* and *Babesia vulpes. Babesia canis* is transmitted by the vector *Dermacentor reticulatus*. Acute *B. canis* infections in dogs are mainly reported from central and eastern European countries and are classified as an emerging disease (Gray et al., 2009; Mierzejewska et al., 2017; Bajer et al., 2022; Diakou, 2024). Different *Babesia* spp. are documented and associated with differences in pathogenicity, leading to subclinical to severe diseases (Solano-Gallego et al., 2008; Leisewitz et al., 2023; Von Hohnhorst et al., 2025b), which was also seen in individual *B. canis* strains (Carcy et al., 2015). *Babesia vogeli*, transmitted by *Rhipicephalus sanguineus*, is mainly found in the Mediterranean area and is mostly linked to subclinical infections (Solano-Gallego et al., 2008; Leisewitz et al., 2023). *Babesia vulpes* infections transmitted by *Ixodes hexagonus* are rarely reported in Spain associated with azotemia and mortality (Camacho et al., 2004).

Rising numbers of autochthonous acute *B. canis* infections were recently described in Germany (Seibert et al., 2022; Schäfer et al., 2023; Weingart et al., 2023). The spatial expansion of the vector tick *D. reticulatus*, increasing import of pets, rising international travel, and changes in land use contribute to the rise in acute autochthonous infections (Gray et al., 2009; Mierzejewska et al., 2017; Bajer et al., 2022; Diakou, 2024). Acute *B*. *canis* infections are characterized by non-specific clinical signs (fever, lethargy, inappetence) ranging from subclinical infections to severe and life-threatening disease, most likely depending on preestablished immunity and levels of parasitemia (Von Hohnhorst et al., 2025b). Thrombocytopenia, anemia, leukopenia, hyperbilirubinemia, increases in liver enzyme activity, azotemia, and hemoglobinuria are the most common laboratory abnormalities (Seibert et al., 2022; Weingart et al., 2023). Most dogs with acute *B. canis* infections show an acute-phase reaction (Von Hohnhorst et al., 2025b). Life-threatening complications like systemic inflammation response syndrome (SIRS), disseminated intravascular coagulopathy (DIC), acute renal failure, and multiple organ dysfunction syndrome (MODS) have also been described (Máthé et al., 2006b; Matijatko et al., 2010; Beletic et al., 2021; Dubova et al., 2023).

Polymerase chain reaction (PCR) on EDTA-blood and/or capillary blood shows high specificity and sensitivity for the detection of deoxyribonucleic acid (DNA) of the causative organism, enables species differentiation, and is therefore considered the gold standard for diagnosis of acute *B. canis* infections (Jefferies et al., 2003; Solano-Gallego et al., 2016). A positive *Babesia* spp. antibody level is interpreted as exposure to the pathogen in the past (Birkenheuer et al., 2003; Maggi et al., 2014). *Babesia* spp. antibody levels are protective against severe and life-threatening disease (Moubri et al., 2018). Acute *B. canis* infections are likely to be associated with less severe clinicopathological abnormalities and acute phase responses in dogs with high antibody levels (Von Hohnhorst et al., 2025b). Seroconversion usually takes place 3–4 weeks after infection (Solano-Gallego et al., 2016).

In general, prognosis is good if diagnosis is prompt and treatment with imidocarb dipropionate is initiated at an early stage of disease. The imidocarb dipropionate dosage recommended by the United States Food and Drug Administration (FDA) is 6.6 mg/kg, applied fortnightly intramuscularly or subcutaneously (FDA, 2026). Complicated courses are described in 12– 41% of affected dogs, and the reported mortality in acute *B. canis* infections varies from < 5% in southwestern Europe to 20% in central and northern Europe (Máthé et al., 2006a; Matijatko et al., 2009; Weingart et al., 2023).

Three studies monitored hematological parameters and acute-phase responses in 10 dogs with experimental (Schetters et al., 2009) as well as natural *B. canis* infections in 50 (Matijatko et al., 2007) and 8 dogs (Spariosu et al., 2021). In these studies, dogs were followed for seven days (Matijatko et al., 2007), 14 days (Schetters et al., 2009), and 15 days (Spariosu et al., 2021). Another study reported fast resolution of thrombocytopenia and leukopenia, but anemia and AST-elevations showed a prolonged time to recover (Máthé et al., 2006a). However, further parameters were not evaluated, and the two dogs included were splenectomised and affected by co-morbidities (Máthé et al., 2006a). In general, the course of disease remains poorly studied and understood in acute natural *B. canis* infections in central Europe.

The present prospective study proposed to monitor clinical signs as well as hematological parameters, biochemistry, coagulation profiles, acute phase proteins, and *Babesia* spp. antibody levels in dogs with naturally derived *B canis* infections after the start of treatment with two injections of imidocarb dipropionate. This extensive data will allow a more comprehensive interpretation of the pathophysiology and the immunological response. We proposed that cytopenias and acute-phase reactions are common in dogs with acute *B. canis* infections at the time of initial presentation, but that these clinical and laboratory abnormalities resolve quickly under treatment in most cases. Furthermore, we proposed that the severity of clinicopathological abnormalities may be inversely correlated with *Babesia* spp. antibody levels in dogs with acute *B. canis* infections.

## 2 Materials and methods

This prospective study included dogs in Germany for which EDTA-blood and serum were sent to the laboratory Laboklin (Bad Kissingen, Germany) between April and November 2024. Veterinarians submitting samples for routine diagnostics to the laboratory Laboklin were asked to fill out a questionnaire if *B. canis* infections were clinically suspected, and to add these questionnaires to the samples’ submission. All diagnostic tests were run at the Laboklin laboratory after overnight shipment. Dogs were included in the study if they tested positive for piroplasms by PCR on EDTA-blood by sequencing of positive PCR results revealing *B. canis* (forward primer: 5′-AAT ACC CAA TCC TGA CAC AGG G-3′; reverse primer: 5′-TTA AAT ACG AAT GCC CCC AAC-3′ (Olmeda et al., 1997)). If this initial PCR testing was positive, a droplet digital PCR (ddPCR, Bio-Rad Laboratories, Inc., Hercules, USA; target: *Bc28.1* gene specific for *B. canis*, forward primer: 5′-GCT ACG TCC GTT GAA GCC-3′ (10 μM), reverse primer: 5′-TCA GCG GAA TAA CGT TCA GC-3′ (10 μM), probe: 5′-FAM-AGC CAG TCG ATC TGC TCC TTT AAG CT-BHQ-3′ (2 μM)) was performed to determine the levels of *B. canis* parasitemia, following the method described by Kivrane et al. (2021).

Dogs were further included if tested serologically negative for *Leishmania infantum* (NovaTec VetLine Leishmania ELISA, Immundiagnostica GmbH, Dietzenbach, Germany) and *Ehrlichia canis* (Ehrlichia ELISA Dog, Afosa, Blankenfelde-Mahlow, Germany) on serum. Additionally, negative antigen-ELISA testing for *Dirofilaria immitis* (FASTTest® HW Antigen, MegaCor GmbH, Hörbranz, Austria), and negative PCR-testing for *Hepatozoon canis* (real-time PCR, target: 16S rDNA), microfilariae, *Anaplasma phagocytophilum* (real-time PCR, target: hsp60 gene), *Anaplasma platys* (real-time PCR, target: groEL gene) and hemotropic *Mycoplasma* spp. (*M. haemocanis*, *M. haematoparvum*; real-time PCR, target: 16S rDNA) were other inclusion criteria, each on EDTA-blood. Dogs were excluded from the study if *Babesia* spp. other than *B. canis* were detected after sequencing. A portion of the study population was previously used to determine Apolipoprotein A-1 levels with another aim to further characterize acute-phase responses in dogs with babesiosis and hemoplasmosis (Von Hohnhorst et al., 2025a).

Three time-points were determined for this study: T0 was defined as the day of diagnosis of acute *B. canis* infections and the day of the first shot of imidocarb dipropionate. No fixed dosage was specified for the dogs included in the study. T1 was considered the day where EDTA-blood and serum were drawn for the second set of analyses and the second imidocarb dipropionate injection was administered. The third set of analyses (T2) were performed with blood drawn 31 days after the first and 14 days after the second imidocarb dipropionate injection. The veterinarians answered questionnaires to elicit anamneses, clinical signs, as well as dosages and time-points of imidocarb dipropionate injections, including the outcomes. Fever was considered if the rectal temperature was >39.0 °C.

Hematology on EDTA-blood included a complete blood count and a manual differential count run on the Sysmex XN-V analyzer (Sysmex Deutschland, Norderstedt, Germany). Each thrombocytopenia <90 G/L was confirmed by manual platelet counting. A hematological scoring system (HES) was designed based on the severity of each hematological abnormality. Anemia, thrombocytopenia, and leukopenia were respectively classified as mild (0.31–0.43 L/L, 91–149 G/L, 4.0–5.9 G/L), moderate (0.20–0.30 L/L, 40–90 G/L, 2.0–3.9 G/L), or marked (< 0.20 L/L, <40 G/L, < 2.0 G/L). If anemia, leukopenia, and/or thrombocytopenia were present, scoring points of 1 (mild), 2 (moderate), or 3 (marked) were added. If pancytopenia was present, an additional 3 points were added. Leukocytosis accounted for 1 additional point in the scoring system. Thus, more marked hematological findings were reflected in a higher HES.

Biochemistry was run on the Cobas 8000 (Roche Deutschland Holding GmbH, Mannheim, Germany) on serum. 1,2-o-dilauryl-rac-glycero-3-glutaric acid-(6′-methylresorufin) ester (DGGR)-lipase, glucose, fructosamine, triglycerides, cholesterol, bilirubin, alkaline phosphatase (AP), alanine aminotransferase (ALT), glutamate dehydrogenase (GLDH), aspartate aminotransferase (AST), creatin kinase (CK), total protein, albumin, globulins, urea, creatinine, phosphorus, magnesium, potassium, sodium, and iron measurements were included. C-reactive protein (CRP) was run on Cobas 8000 using the Gentian Canine CRP Immunoassay (Gentian Diagnostics, Moos, Norway) on serum. To calculate the urea:creatinine ratio with the aim to differentiate between prerenal and renal azotemia, both parameters were first converted to the unit mg/dl using the conversion factors ×6.0060 (urea) and ×0.0113 (creatinine), respectively. Urea was then divided by creatinine to derive the ratio.

Additionally, antibody-ELISAs for *Babesia* spp. (*Babesia* ELISA Dog, Afosa, Blankenfelde-Mahlow, Germany) with sensitivity up to 96.3% and specificity up to 100%, validated on 287 dogs by the manufacturer, were performed on serum. Direct Coombs’ testing on EDTA-blood was carried out in the Laboklin laboratory. If citrate blood was available, coagulation status was assessed through measurement of the prothrombin time (PT), activated partial thromboplastin time (aPTT), thrombin time (TT) and fibrinogen (STA Compact Max3, DIAGNOSTICA STAGO, Asnieres sur Seine, France).

The results were statistically analyzed using SPSS (version 30.0, IBM); *P* ≤ 0.05 was considered statistically significant. Kruskal-Wallis testing including Bonferroni corrections was performed to compare laboratory parameters between the three different time-points of the study. Spearman’s correlation analysis was used to correlate the concentrations of selected key laboratory parameters (hematocrit, white blood cells, platelets, CRP, fibrinogen, total protein, albumin, creatinine, bilirubin, iron, *Babesia* spp. antibody levels, levels of parasitemia). Correlations with ρ ≥ 0.5 were considered as strong, those with ρ = 0.300–0.499 as moderate, and those with ρ = 0.100–0.299 as mild (Cohen, 1988). Repeated measures ANOVA with Greenhouse-Geisser correction, including Bonferroni-adjusted *post-hoc* analysis, was applied for selected key laboratory parameters. To address the early treatment response in the first 14 days, differences of the selected key laboratory parameters, hematocrit, white blood cells, and platelets as well as bilirubin, fibrinogen, and CRP concentrations between T0 and T1 were calculated for dynamic analysis and correlated to the initial *Babesia* spp. antibody levels, pathogen quantification, and dosage of imidocarb dipropionate by Spearman’s correlation analysis, as indicated above.

## 3 Results

### 3.1 Study population

This prospective study included 23 dogs living in Germany. Information about signalment and anamnesis was available for all 23 dogs. Ten dogs were male (43%, all intact), and the remaining 13 were female (57%, 2/13 spayed (15%)). The median age was 5.2 years, ranging from 1 to 14 years. Eleven of the 23 dogs (48%) were mixed breeds. The remaining dogs were Labrador Retrievers (4/23; 17%), Rottweilers (2/23; 9%), Leonberger (1/23; 4%), Golden Retriever (1/23; 4%), German Spaniel Dog (1/23; 4%) and a Greater Swiss Mountain dog (1/23; 4%). Co-morbidities were reported in 3 of the 23 dogs (13%) and included atopic dermatitis in two dogs and pulmonary stenosis in one dog. One of the two dogs with atopic dermatitis was under therapy with oclacitinib, whereas the dog with pulmonary stenosis was receiving atenolol.

Pre-medication with corticosteroids was reported in three dogs (13%). The first dog received prednisolone with a dosage of 1 mg/kg, starting a day prior to the day of the *B. canis* diagnosis. The second dog was administered dexamethasone in an unknown dosage one month prior to diagnosis for an unknown reason. Neither the substance nor the dosage nor the moment of administration was known for the third dog.

In the two weeks preceding the diagnosis, owners noted tick infestation in 16 of the 23 dogs (70%). Upon clinical examination by the veterinarian, 15 dogs (65%) showed tick infestation. Information about stays abroad was available for all 23 dogs. Sixteen dogs (70%) did not have any stays abroad. Of those 16 dogs, ten samples (63%) were sent in from the federal states of Brandenburg, five (31%) from Saxony-Anhalt and one (6%) from Saxony. Stays abroad from Germany were mentioned in 7 of the 23 dogs (30%). Two out of 23 dogs (9%) had been imported, and another two dogs (9%) were imported and travelled afterwards to other countries (import from Romania: *n* = 2; Hungary: *n* = 1; and Italy: *n* = 1). Three dogs (13%) had a travel anamnesis (Croatia: *n* = 1; the Netherlands: *n* = 1; unknown: *n* = 1).

In seven of 17 dogs (41%), information was available about the federal states the owners lived in, and in all seven cases, the federal states of the veterinary practices coincided with the federal states in which the owner lived.

The exact dates for T0, T1, and T2 were known in all dogs. The median for T1 was 15 days after T0 (range: 13–30 days), and 32 days after T0 for T2 (range: 27–47 days). The median time that elapsed between T1 and T2 was 17 days (range: 15–26 days).

### 3.2 Clinical signs and general examination

The main clinical signs reported by the owners included lethargy in 19 of the 23 dogs (83%), hyporexia/inappetence in 13 of 19 dogs (68%), and bloody/darkened urine in 3 of 14 dogs (21%). Gastrointestinal signs such as vomitus were seen in 1 of 17 dogs (6%). The results of the general examination at T0 were available in all 23 dogs. Of the 23 dogs included, two (9%) showed no clinical signs and had an unremarkable physical examination, both of which were imported. Lethargy was reported in 19 of the 23 (83%) dogs and fever in 15 of the 23 dogs (65%; median 39.4 °C; range 37.3–40.2 °C). Pale mucous membranes were observed in 10/23 dogs (43%). Capillary refill time was prolonged (≥ 2 s) in 9 of the 23 dogs (39%). Palpation of peripheral lymph nodes revealed no lymph adenomegaly in all 23 dogs. At T1 and T2, results of clinical examination were available in five and six dogs, respectively. These clinical examinations were classified as unremarkable.

### 3.3 Hematological and biochemical findings

**Fig. 1:**
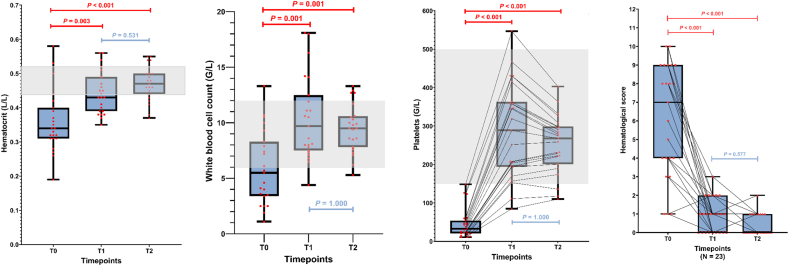
Hematocrit, white blood cell count, platelet count, and hematological score (HES) in 23 dogs with an acute *Babesia canis* infection sorted by three time-points (T0, T1, and T2). Grey rectangles indicate the reference ranges.

Complete hematological analyses were available for all 23 dogs at each time-point and are summarized in Table 1. Thrombocytopenia was predominantly marked (13/23; 57%) and anemia was most often mild (15/19; 79%) (Supplementary Table S1, Fig. 1). At T0, the HES was the highest of all time-points (median: 7, range: 1–10) with significant decrease compared to T1 (median: 0, range: 0–3) and T2 (median: 0, range: 0–1) (*P* < 0.001, each). No significant impact was noted between T1 and T2 (*P* = 0.577). Significant increases were observed in hematocrit, white blood cell counts, and platelet counts comparing T0 to T1 as well as T2 (*P* < 0.003, respectively), but not between T1 and T2 (*P* > 0.05, respectively) (Table 2, Supplementary Table S1, Fig. 1).

**Table 1:** Main laboratory abnormalities in 23 dogs with acute *Babesia canis* infections at the time of diagnosis and first injection of imidocarb dipropionate, as well as changes in laboratory parameters by T1 (second imidocarb dipropionate injection, median time to T0 15 days) and T2 (checkup, median time to T0 32 days) compared to T0, respectively.

Laboratory abnormality	*n*/*N* (%)	Significant change from T0 to T1	Significant change from T1 to T2	Significant change from T0 to T2
**Hematology**^a^				
Pancytopenia	11/23 (48)	–	–	–
Thrombocytopenia	23/23 (100)	Yes	No	Yes
Anemia	19/23 (83)	Yes	No	Yes
Leukopenia	13/23 (57)	Yes	No	Yes
**Biochemistry**^b^				
Elevated C-reactive protein^c^	20/23 (87)	Yes	No	Yes
Hyperbilirubinemia	18/23 (78)	Yes	No	Yes
Elevated asparatate transferase	17/23 (74)	Yes	No	Yes
Elevated creatine kinase	14/23 (61)	Yes	No	Yes
Decreased iron	14/23 (61)	Yes	No	Yes
Elevated alkaline phosphatase	12/23 (52)	No	No	Yes
Elevated urea	12/23 (52)	No	No	No
Decreased total protein	11/23 (48)	Yes	No	Yes
Increased 1,2-o-dilauryl-rac-glycero-3-glutaric acid-(6′-methylresorufin) ester (DGGR) lipase	9/23 (39)	Yes	No	No
***Babesia* spp. antibody levels**^d^				
Negative *Babesia* spp. antibody levels	20/23 (87)	Yes	No	Yes

a Sysmex XN-V analyzer, Sysmex Deutschland, Norderstedt, Germany (on EDTA-blood).

b Cobas 8000, Roche Deutschland Holding GmbH, Mannheim, Germany (on serum).

c Gentian Canine CRP Immunoassay, gentian diagnostics, Moos, Norway (on serum).

d *Babesia* ELISA Dog, Afosa, Blankenfelde-Mahlow, Germany (on serum).

**Table 2:** Decrease and increase in laboratory parameters with statistically significant differences between the three time-points T0, T1, and T2, in 23 dogs with acute *Babesia canis* infections according to the reference intervals of the Laboklin laboratory.

Parameter (reference interval)^a^	T0 (first diagnosis and first imidocarb dipropionate injection)		T1 (second imidocarb dipropionate injection, median time to T0 15 days)		T2 (checkup, median time to T0 32 days)		Significance of differences in parameter concentrations^b^
	Decreased (%)	Increased (%)	Decreased (%)	Increased (%)	Decreased (%)	Increased (%)	
**Hematology**^c^							
Hematocrit (0.44–0.52 L/L)	19/23 (83)	2/23 (9)	12/23 (52)	0/23 (0)	3/23 (13)	2/23 (9)	T0 *vs* T1: *P* = 0.003
							T0 *vs* T2: *P* < 0.001
Reticulocytes (< 110 μL)	0/23 (0)	0/23 (0)	0/23 (0)	12/23 (52)	0/23 (0)	3/23 (13)	T0 *vs* T1: *P* < 0.001
							T0 *vs* T2: *P* < 0.001
White blood cells (6.0–12.0 G/L)	13/23 (57)	1/23 (4)	5/23 (22)	5/23 (22)	0/23 (0)	4/23 (17)	T0 *vs* T1: *P* = 0.010
							T0 *vs* T2: *P* < 0.001
Segmented neutrophilic granulocytes (3.0–11.5 G/L)	10/23 (43)	0/23 (0)	1/23 (4)	1/23 (4)	1/23 (4)	0/23 (0)	T0 *vs* T1: *P* < 0.001
							T0 *vs* T2: *P* = 0.002
Lymphocytes (1.0–3.6 G/L)	11/23 (48)	1/23 (4)	0/23 (0)	3/23 (13)	0/23 (0)	3/23 (13)	T0 *vs* T1: *P* = 0.001
							T0 *vs* T2: *P* < 0.001
Eosinophilic granulocytes (0.04–0.60 G/L)	1/23 (4)	16/23 (70)	2/23 (9)	10/23 (43)	1/23 (4)	13/23 (57)	T0 *vs* T1: *P* < 0.001
							T0 *vs* T2: *P* < 0.001
Thrombocytes (150–500 G/L)	23/23 (100)	0/23 (0)	2/23 (9)	1/23 (4)	3/23 (13)	0/23 (0)	T0 *vs* T1: *P* < 0.001
							T0 *vs* T2: *P* < 0.001
**Biochemistry**^d^							
Alkaline phosphatase (< 147 U/L)	0/23 (0)	12/23 (52)	0/23 (0)	4/23 (17)	0/23 (0)	3/23 (13)	T0 *vs* T2: *P* = 0.003
Aspartate aminotransferase (< 51 U/L)	0/23 (0)	17/23 (74)	0/22 (0)	0/22 (0)	0/23 (0)	1/23 (4)	T0 *vs* T1: *P* < 0.001
							T0 *vs* T2: *P* < 0.001
Creatine kinase (< 200 U/L)	0/23 (0)	14/23 (61)	0/23 (0)	0/23 (0)	0/23 (0)	4/23 (17)	T0 *vs* T1: *P* < 0.001
							T0 *vs* T2: *P* = 0.001
Bilirubin (< 3.4 μmol/L)	0/23 (0)	18/23 (78)	0/22 (0)	1/22 (5)	0/22 (0)	0/22 (0)	T0 *vs* T1: *P* < 0.001
							T0 *vs* T2: *P* < 0.001
Total protein (54–75 g/L)	11/23 (48)	0/23 (0)	0/23 (0)	0/23 (0)	1/23 (4)	0/23 (0)	T0 *vs* T1: *P < *0.001
							T0 *vs* T2: *P* < 0.001
Albumin (25–44 g/L)	2/23 (9)	0/23 (0)	0/23 (0)	0/23 (0)	0/23 (0)	0/23 (0)	T0 *vs* T1: *P* = 0.007
							T0 *vs* T2: *P* < 0.001
1,2-o-dilauryl-rac-glycero-3-glutaric acid-(6′-methylresorufin) ester lipase (< 120 U/L)	0/23 (0)	9/23 (39)	0/22 (0)	3/22 (14)	0/23 (0)	1/23 (4)	T0 *vs* T1: *P* = 0.034
Calcium (2.3–3.0 mmol/L)	12/23 (52)	0/23 (0)	1/23 (4)	1/23 (4)	0/23 (0)	0/23 (0)	T0 *vs* T1: *P* = 0.003
							T0 *vs* T2: *P* < 0.001
Iron (15–45 μmol/L)	14/23 (61)	0/23 (0)	2/22 (9)	3/22 (14)	1/23 (4)	1/23 (4)	T0 *vs* T1: *P* = 0.046
							T0 *vs* T2: *P* = 0.002
Potassium (3.5–5.1 mmol/L)	1/23 (4)	2/23 (9)	0/22 (0)	5/22 (23)	0/23 (0)	5/23 (22)	T0 *vs* T1: *P* < 0.001
							T0 *vs* T2: *P* < 0.001
C-reactive protein (< 15 mg/L)^e^	0/23 (0)	20/23 (87)	0/23 (0)	1/23 (4)	0/23 (0)	1/23 (4)	T0 *vs* T1: *P* < 0.001
							T0 *vs* T2: *P* < 0.001
**Coagulation status**^f^							
Thrombin time (10.0–18.3 s)	3/19 (16)	0/19 (0)	0/19 (0)	0/19 (0)	0/19 (0)	1/19 (5)	T0 *vs* T1: *P* < 0.001
							T0 *vs* T2: *P* = 0.002
Fibrinogen (130–310 mg/dL)	1/19 (5)	16/19 (84)	0/19 (0)	7/19 (37)	1/19 (5)	1/19 (5)	T0 *vs* T1: *P* = 0.003
							T0 *vs* T2: *P < *0.001
***Babesia* spp. antibody levels**^g^	**Negative (%)**	**Positive (%)**	**Negative (%)**	**Positive (%)**	**Negative (%)**	**Positive (%)**	**Comparison: *P*-value**
Antibody level (< 19.0 technical units)	20/23 (87)	3/23 (13)	1/23 (4)	22/23 (96)	2/23 (9)	21/23 (91)	T0 *vs* T1: *P* < 0.001
							T0 *vs* T2: *P = *0.001

a Reference intervals according to the Laboklin laboratory (Bad Kissingen, Germany).

b Kruskal-Wallis test including Bonferroni correction.

c Sysmex XN-V analyzer, Sysmex Deutschland, Norderstedt, Germany (on EDTA-blood).

d Cobas 8000, Roche Deutschland Holding GmbH, Mannheim, Germany (on serum).

e Gentian Canine CRP Immunoassay, gentian diagnostics, Moos, Norway (on serum).

f STA Compact Max3, DIAGNOSTICA STAGO, Asnieres sur Seine, France (on citrated blood).

g *Babesia* ELISA Dog, Afosa, Blankenfelde-Mahlow, Germany (on serum).

Initially at T0, all dogs (100%) were affected by thrombocytopenia (median: 33 G/L; standard deviation (SD): 38.1 G/L). By T1, the platelet count had normalized in 21 of the 23 dogs (91%) (median: 289 G/L; SD: 124.3 G/L) (Table 2, Supplementary Table S1). One of the remaining two dogs had marked thrombocytopenia at T0 (13 G/L), which evolved into a mild thrombocytopenia (111 G/L) by T1. In another dog, a drop in thrombocytes was observed between the two time-points: the initially mild thrombocytopenia (123 G/L) became moderate (85 G/L) at T1. At T2, thrombocytopenia was seen in 3 of the 23 dogs (13%) and was mild in each case. Both dogs with thrombocytopenia at T1 were still classified as thrombocytopenic at T2. The third dog initially had mild thrombocytopenia (149 G/L) which normalized by T1 (204 G/L) but reappeared at T2 (136 G/L).

The hematocrit rose significantly comparing T0 with T1 (*P* = 0.003) and T0 with T2 (*P* < 0.001) (Table 2, Supplementary Table S1, Fig. 1). Mild anemia was still present in 12 out of 19 initially affected dogs (63%) at T1. By that time-point, the four moderate/marked anemia cases at T0 became either mild or completely normalized. At T2, 3 of the 19 initially affected dogs (16%) were still affected by mild anemia. At T0, no dogs showed increased reticulocytes. Fourteen dogs showed reticulocytosis at either T1 (11/14; 79%), T2 (2/14; 14%), or both (1/14; 7%). Thirteen out of these 14 dogs (93%) were anemic at T0, with one dog showing reticulocytosis without having concurrent anemia. Six of the 19 initially anemic dogs (32%) showed no reticulocytosis at T1 or T2. The differences between T0 and T1, as well as T0 and T2 were significant (*P* < 0.001 each).

A significant raise in leukocytes was demonstrated comparing T0 with T1 and T0 with T2, respectively (*P* = 0.01, each) (Table 2, Supplementary Table S1, Fig. 1). Leukopenia, initially affecting 13 of the 23 dogs (57%), resolved in eight cases (62%) by T1. At that time-point, leukopenia persisted in one dog but evolved from moderate at T0 (2.5 G/L) to mild at T1 (4.4 G/L). Leukocytosis was recorded in 1 of the 23 dogs (4%) at T0 compared to 5 of the 23 dogs (22%) at T1. At T2, the last leukopenia had resolved and merely 4 of the 23 dogs (17%) still showed mild leukocytosis.

Comparison of the differential blood count between the three time-points (T0 and T1, T0 and T2) led to significant increases in lymphocytes (*P = *0.001 and *P < *0.001, respectively), eosinophilic granulocytes (*P < *0.001 and *P* < 0.001, respectively) and segmented neutrophils (*P* < 0.001 and *P* = 0.001, respectively) (Table 2, Supplementary Table S1).

The most common biochemical findings at T0 are summarized in Table 1. Out of these nine dogs with elevated DGGR-lipase, four (44%) had prolonged capillary refill time upon the clinical examination at T0.

**Fig. 2:**
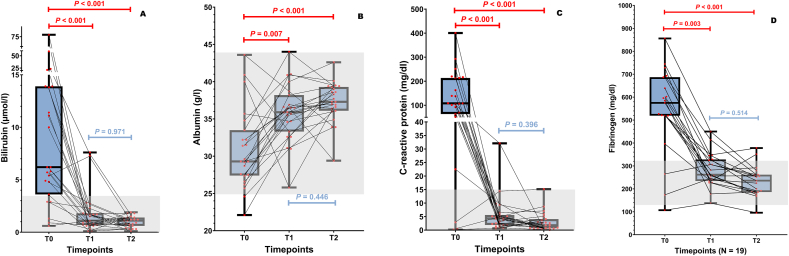
Bilirubin (**A**), albumin (**B**), C-reactive protein (**C**), and fibrinogen concentrations (**D**) in 23 dogs with an acute *Babesia canis* infection sorted by three time-points (T0, T1, and T2). Connecting lines represent the blood test timelines in individual dogs. Grey rectangles indicate the reference ranges.

Hyperbilirubinemia was one of the most common abnormalities in the initial biochemical analysis, affecting 78% of the dogs at T0. Seventeen out of 18 dogs (94%) had concurrent anemia. In only 1 of the 18 dogs (6%), hyperbilirubinemia had not normalized by T1; but it had significantly decreased (from 77.9 μmol/l at T0 to 7.3 μmol/l at T1). By T2, bilirubin values were physiological in all 23 dogs. Differences in the bilirubin values were significant between T0 and T1 as well as T1 and T2 (*P* < 0.001 each), but not between T1 and T2 (Table 2, Supplementary Table S1, Fig. 2).

Decreased iron values were found in 61% of the 22 dogs at T0. By T1, 86% of the initially decreased values had normalized, and three dogs (14%) showed increased values. By T2, 21 of the 23 dogs (91%) had physiological iron values, while one dog (4%) had increased values. Merely one dog (4%), which had physiological iron values at T0 and T1, showed a mild decrease in iron concentration at T2.

Azotemia was manifested through elevated urea (12/23 dogs; 52%) and creatinine (7/23 dogs; 30%). For all seven dogs with elevated urea and elevated creatinine at T0, the urea:creatinine ratio exceeded 15. At T1, urea had normalized in 10 out of 12 dogs (83%), and creatinine elevation had subsided in 6 out of 7 dogs (86%). The only dog having increased creatinine at T1, however, showed a significant decrease in values (from 997 μmol/l at T0 to 144 μmol/l at T1). At T2, urea elevation persisted in 2 out of 12 dogs (17%) with increased values at T1. Increases in creatinine, on the other hand, had resolved in all cases at T2.

Decreased values of total protein, initially found in 48% of cases, had resolved by T1 in all dogs, and affected only one individual (4%) at T2. Hypoalbuminemia, affecting 2 out of 23 dogs (9%) at T0, resolved by T1 in both cases. CRP showed a significant decrease between T0 and T1 (*P* < 0.001) as well as T0 and T2 (*P* < 0.001) (Table 1, Fig. 2). A most often marked increase in CRP values was noted in 87% of the study cohort by T0. By T1, one of the 20 dogs (5%) still showed a mild increase, while all other elevations (19/20 dogs; 95%) had subsided. By T2, only one elevation was noted in a dog with an initial increase but normalization by T1 (Table 2, Supplementary Table S1, Fig. 2).

The differences in fibrinogen values were significant between T0 and T1 (*P* = 0.003) as well as T0 and T2 (*P* < 0.001). At T0, fibrinogen was decreased in 5% (1/19) and increased in 84% (16/19) of the dogs. The initially decreased value normalized by T1. Fibrinogen increased in 37% (7/19) of the dogs and subsided by that time-point. By T2 fibrinogen elevations had normalized in 95% (18/19) of the dogs. The dog initially showing a decreased fibrinogen value, which normalized by T1, showed another decrease at T2 (Table 2, Supplementary Table S1, Fig. 2).

Coagulation status was further assessed through measurement of prothrombin time (PT), activated partial thromboplastin time (aPTT), and thrombin time (TT) in 19 out of 23 dogs (83%). PT was physiological in each dog at each time-point. The aPTT was prolonged in 5 of the 19 dogs (26%) at T0 but was physiological at T1 in all dogs. By T2, merely 1 of the 19 dogs (5%), which was not affected initially, showed a prolonged aPTT. Thrombin time (TT) values were decreased in 3 of the 19 dogs (16%) at T0 but were physiological in all dogs at T1 and T2, except for one dog (5%) with elevated TT at T2. These differences were statistically significant between T0 and T1 (*P* < 0.001) as well as T0 and T2 (*P* = 0.002) (Table 2, Supplementary Table S1).

The Coombs’ test was positive in 6 of the 23 dogs (26%) at T0. Of these six dogs, five (83%) were anemic at T0 (mild anemia: *n* = 3; moderate anemia: *n* = 2). At T1 and T2 time-points, all Coombs’ tests performed in all 23 dogs were negative.

### 3.4 *Babesia* spp. antibodies and levels of *B. canis* parasitemia

PCR testing for *B. canis* was positive in all 23 dogs at T0. The levels of parasitemia were measured in all 23 dogs and ranged from 1.7 × 10^2^ parasites to 102 × 10^6^ parasites/ml EDTA blood (median: 836 × 10^4^ parasites/ml, interquartile range: 2129 × 10^4^ parasites/ml).

**Fig. 3:**
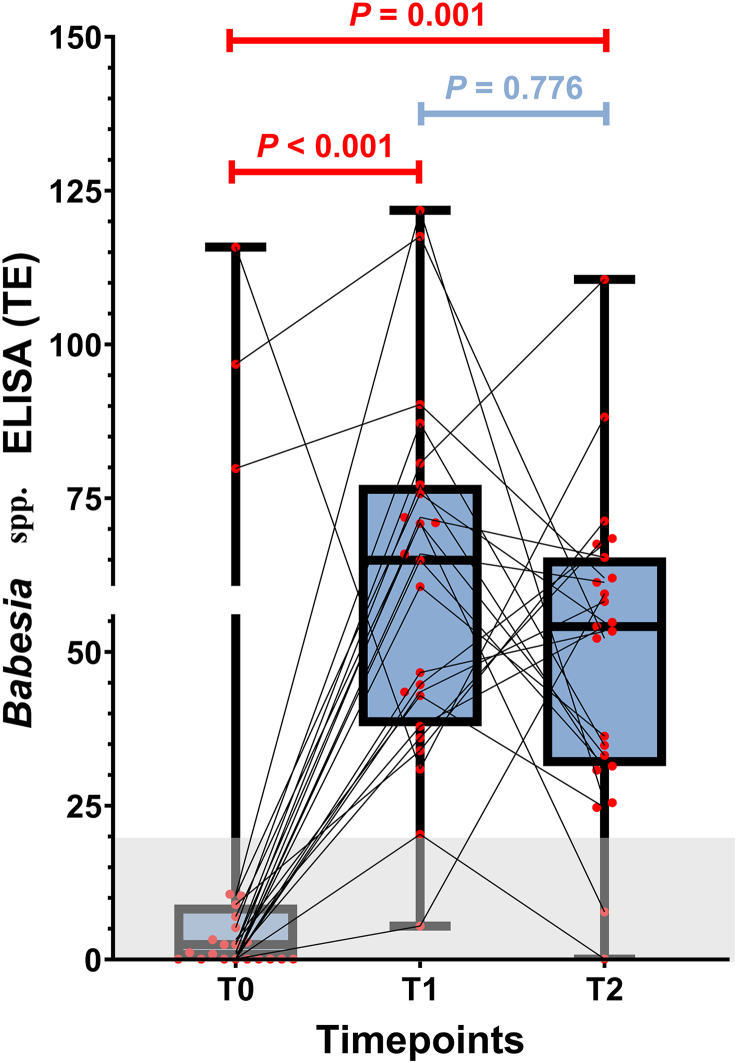
*Babesia* spp. antibody levels on serum in 23 dogs with an acute *Babesia canis* infection sorted by three time-points (T0, T1, and T2). Connecting lines represent the blood test timelines in individual dogs. The grey rectangle indicates the reference range.

*Babesia* spp. antibody testing was performed in all 23 dogs at all three time-points. Differences in antibody levels were significant between T0 and T1 (*P* < 0.001) as well as T0 and T2 (*P* = 0.001). Upon initial analysis, 20 of the 23 dogs (87%) were serologically negative (Table 2, Supplementary Table S1, Fig. 2). The three serologically positive dogs were all imported individuals and had *Babesia* antibody levels of 99.8 technical units (TE), 115.8 TE, and 94.8 TE, respectively (Fig. 3). In 2 of the 3 dogs, antibody levels decreased continuously by T1 and T2. The dog with the highest antibody level at T0 (115.8 TE) showed a slight increase by T1 (121.8 TE) and a mild decrease by T2 (110.6 TE). Of the import group, only 1 out of 4 dogs (25%) was serologically negative. The two dogs that had travelled and the 16 dogs without stays abroad were also serologically negative at T0. Of these 20 serologically negative dogs, 18 (90%) became serologically positive by T1. The antibody level in these 18 dogs at T1 decreased in 15 dogs (83%) between T1 and T2, but all remained positive at that time-point. The two remaining dogs stayed serologically negative, even at T2 (Table 2, Supplementary Table S1, Fig. 3).

Interestingly, all three dogs with positive *Babesia* spp. antibody levels were imported from Romania (*n* = 2) and Hungary (*n* = 1) to Germany and showed the lowest *B. canis* parasitemia in the study (170 parasites/ml, 710 parasites/ml, and 6600 parasites/ml, respectively). All three dogs were unremarkable in clinical examination and PCR testing was requested by the veterinarians due to high antibody levels and mild thrombocytopenia (123 G/L, 126 G/L, and 149 G/L, respectively) in each dog. No further hematological abnormalities were noted, and CRP was in the reference interval in all three dogs (0.2 mg/L, 0.5 mg/L, and 2.9 mg/L, respectively).

### 3.5 Correlation analysis, repeated measures ANOVA

Results of the correlation analysis covering all timeframes and for T0 are presented in Table 3, Table 4, respectively. *Babesia* spp. antibody levels showed strong positive correlations with hematocrit, platelets, and total protein concentrations (Table 3). Moderate positive correlations were detected with white blood cells, creatinine and iron concentrations. Strong negative correlations were detected with CRP, fibrinogen, and bilirubin (Table 3). At T0, *Babesia* spp. antibody levels were moderately positively correlated with total protein and negatively correlated with bilirubin concentrations (Table 4). The level of *B. canis* parasitemia showed strong negative correlations to the *Babesia* spp. antibody levels and the white blood cell count, as well as a strong positive correlation with the bilirubin concentrations (Table 4). The rectal temperature measured in each of the 23 dogs at T0 showed strong negative correlations with the white blood cell count and the iron concentrations, as well as a strong positive correlation with the levels of *B. canis* parasitemia and a moderate positive correlation with the CRP concentrations (Table 4).

**Table 3:** Correlation analysis (Spearman’s ρ) showing moderate (> 0.300–0.499) to strong (≥ 0.500) correlations of selected key laboratory parameters in 23 dogs with acute *Babesia canis* infections at the time of diagnosis (T0), the time of the second imidocarb dipropionate injection (T1), and two weeks after T1 (T2).

		Hematocrit	White blood cells	Platelets	C-reactive protein	Fibrinogen	Total protein	Albumin	Creatinine	Bilirubin	Iron
White blood cells	ρ	**0.358**									
	*P*	**0.003∗∗**									
	*n*	69									
Platelets	ρ	**0.410**	**0.415**								
	*P*	**<0.001∗∗∗**	**<0.001∗∗∗**								
	*n*	69	69								
C-reactive protein	ρ	**−0.724**	**−0.421**	**−0.542**							
	*P*	**<0.001∗∗∗**	**<0.001∗∗∗**	**<0.001∗∗∗**							
	*n*	69	69	69							
Fibrinogen	ρ	**−0.645**	**−0.500**	**−0.535**	**0.727**						
	*P*	**<0.001∗∗∗**	**<0.001∗∗∗**	**<0.001∗∗∗**	**<0.001∗∗∗**						
	*n*	57	57	57	57						
Total protein	ρ	**0.580**	**0.332**	**0.630**	**−0.531**	**−0.518**					
	*P*	**<0.001∗∗∗**	**0.005∗∗**	**<0.001∗∗∗**	**<0.001∗∗∗**	**<0.001∗∗∗**					
	*n*	69	69	69	69	57					
Albumin	ρ	0.113	−0.048	0.145	0.010	0.081	0.192				
	*P*	0.354	0.693	0.235	0.933	0.549	0.113				
	*n*	69	69	69	69	57	69				
Creatinine	ρ	**0.795**	**0.343**	**0.411**	**−0.731**	**−0.682**	**0.585**	0.045			
	*P*	**<0.001∗∗∗**	**0.004∗∗**	**<0.001∗∗∗**	**<0.001∗∗∗**	**<0.001∗∗∗**	**<0.001∗∗∗**	0.712			
	*n*	69	69	69	69	57	69	69			
Bilirubin	ρ	**−0.573**	**−0.434**	**−0.544**	**0.654**	**0.589**	**−0.374**	−0.020	**−0.564**		
	*P*	**<0.001∗∗∗**	**<0.001∗∗∗**	**<0.001∗∗∗**	**<0.001∗∗∗**	**<0.001∗∗∗**	**0.002∗∗**	0.872	**<0.001∗∗∗**		
	*n*	67	67	67	67	55	67	67	67		
Iron	ρ	**0.438**	**0.385**	**0.308**	**−0.535**	**−0.523**	**0.352**	−0.052	**0.588**	**−0.400**	
	*P*	**<0.001∗∗∗**	**0.001∗∗∗**	**0.011∗**	**<0.001∗∗∗**	**<0.001∗∗∗**	**0.003∗∗**	0.673	**<0.001∗∗∗**	**0.001∗∗∗**	
	*n*	68	68	68	68	56	68	68	68	67	
*Babesia* spp. antibody levels	ρ	**0.563**	**0.461**	**0.542**	**−0.553**	**−0.723**	**0.580**	0.165	**0.447**	**−0.541**	**0.346**
	*P*	**<0.001∗∗∗**	**<0.001∗∗∗**	**<0.001∗∗∗**	**<0.001∗∗∗**	**<0.001∗∗∗**	**<0.001∗∗∗**	0.176	**<0.001∗∗∗**	**<0.001∗∗∗**	**0.004∗∗**
	*n*	69	69	69	69	57	69	69	69	67	68

*Abbreviations*: ρ, Spearman’s correlation coefficient; *P*, significance value (two-sided); *n*, number of dogs.

*Note*: Statistically significant associations are highlighted in bold with *P*-values indicated with asterisks (∗*P* < 0.05; ∗∗*P* < 0.01; ∗∗∗*P* < 0.001).

**Table 4:** Correlation analysis (Spearman’s ρ) showing moderate (> 0.300–0.499) to strong (≥ 0.500) correlations of selected key laboratory parameters in 23 dogs with acute *Babesia canis* infections at the time of diagnosis (T0).

		Hematocrit	White blood cells	Platelets	C-reactive protein	Fibrinogen	Total protein	Albumin	Creatinine	Bilirubin	Iron	*Babesia* antibody levels	Levels of parasitemia
White blood cells	ρ	0.161											
	*P*	0.463											
	*n*	23											
Platelets	ρ	0.380	0.323										
	*P*	0.073	0.132										
	*n*	23	23										
C-reactive protein	ρ	−0.105	**−0.472**	**−0.457**									
	*P*	0.634	**0.023∗**	**0.028∗**									
	*n*	23	23	23									
Fibrinogen	ρ	−0.090	−0.397	−0.396	**0.805**								
	*P*	0.715	0.092	0.093	**<0.001∗∗∗**								
	*n*	19	19	19	19								
Total protein	ρ	**0.593**	0.123	**0.575**	−0.048	−0.139							
	*P*	**0.003∗∗**	0.576	**0.004∗∗**	0.828	0.571							
	*n*	23	23	23	23	19							
Albumin	ρ	0.383	−0.066	0.195	0.098	0.284	**0.427**						
	*P*	0.072	0.764	0.372	0.657	0.238	**0.042∗**						
	*n*	23	23	23	23	19	23						
Creatinine	ρ	**0.707**	0.095	**0.473**	−0.076	−0.197	**0.761**	0.340					
	*P*	**<0.001∗∗∗**	0.668	**0.023∗**	0.732	0.420	**<0.001∗∗∗**	0.113					
	*n*	23	23	23	23	19	23	23					
Bilirubin	ρ	−0.197	−0.402	−0.278	0.325	**0.494**	−0.318	**0.517**	−0.217				
	*P*	0.368	0.057	0.199	0.130	**0.032∗**	0.139	**0.011∗**	0.321				
	*n*	23	23	23	23	**19**	23	23	23				
Iron	ρ	0,024	**0.717**	0.377	**−0.558**	**−0.615**	0.093	−0.063	−0.035	−0.254			
	*P*	0.914	**<0.001∗**	0.076	**0.006∗∗**	**0.005∗∗**	0.673	0.774	0.875	0.243			
	*n*	23	23	23	23	19	23	23	23	23			
*Babesia* antibody levels	ρ	0,348	0.390	0.346	−0.114	−0.130	**0.466**	0.117	0.299	**−0.422**	0.278		
	*P*	0.104	0.066	0.106	0.604	0.595	**0.025∗**	0.594	0.166	**0.045∗**	0.199		
	*n*	23	23	23	23	19	23	23	23	23	23		
Levels of parasitemia	ρ	−0.342	**−0.582**	−0.225	0.308	0.390	−0.262	−0.056	0.269	**0.773**	−0.359	**−0.513**	
	*P*	0.110	**0.004∗∗**	0.303	0.152	0.099	0.226	0.798	0.214	**<0.001∗∗∗**	0.093	**0.012∗**	
	*n*	23	23	23	23	19	23	23	23	23	23	23	
Rectal temperature	ρ	−0.184	**−0.858**	−0.353	**0.481**	0.222	−0.082	0.058	−0.066	0.276	**−0.603**	−0.354	**0.581**
	*P*	0.401	**<0.001∗∗∗**	0.098	**0.020∗**	0.360	0.711	0.794	0.765	0.202	**0.002∗∗**	0.097	**0.004∗∗**
	*n*	23	23	23	23	19	23	23	23	23	23	23	23

*Abbreviations*: ρ, Spearman’s correlation coefficient; *P*, significance value (two-sided); *n*, number of dogs.

*Note*: Statistically significant associations are highlighted in bold with *P*-values indicated with asterisks (∗*P* < 0.05; ∗∗*P* < 0.01; ∗∗∗*P* < 0.001).

Repeated measures ANOVA revealed the highest partial η^2^ values for key laboratory parameters with significant differences (*P* < 0.001, respectively) between the different time-points for thrombocytes (0.745), fibrinogen (0.720), CRP (0.643), and hematocrit (0.610) (Table 5).

**Table 5:** Repeated measures ANOVA with Greenhouse-Geisser correction, including the three time-points T0, T1, and T2 in 23 dogs with acute *Babesia canis* infections, with Bonferroni-adjusted *post-hoc* analysis for selected key hematological, biochemical, and coagulation parameters as well as *Babesia* spp. antibody levels.

Parameter	*F* (*df* Zähler, *df* Nenner)	*P*	*ᶯ*^2^	T0-T1 (*P*, *M*_diff_, [95% CI])	T1-T2 (*P*, *M*_diff_, [95% CI])	T0-T2 (*P*, *M*_diff_, [95% CI])
**Hematology**						
Hematocrit	34.38 (0.16, 0.10)	<0.001	0.610	<0.001, −0.09, [−0.12, −0.05]	0.031, −0.03, [−0.05, −0.002]	<0.001, −0.11, [−0.16, −0.07]
White blood cells	19.34 (1.55, 34.02)	<0.001	0.468	<0.001, −4.46, [−6.91, −2.02]	0.582, 0.80, [−0.75, 2.35]	<0.001, −3.66, [−5.51, −1.81]
Thrombocytes	64.304 (1.29, 28.31)	<0.001	0.745	<0.001, −242.17, [−320.71, −163.63]	0.130, 34.04, [−7.12, 75.21]	<0.001, −208.13, [−262.02, −154.24]
**Biochemistry**						
Alkaline phosphatase	1.44 (1.12, 24.71)	0.246	0.061	1.000, 8.54, [−87.27, 104.35]	0.025, 36.41, [3.87, 68.96]	0.415, 44.96, [−30.80, 120.71]
Aspartate aminotransferase	24.39 (1.02, 21.37)	<0.001	0.537	<0.001, 88.36, [44.05, 132.68]	0.272, −3.67, [−9.06, 1.72]	<0.001, 84.69, [38.14, 131.24]
Alanine aminotransferase	0.14 (1.22, 24.48)	0.758	0.007	1.000, 0.10, [−32.66, 32.86]	1.000, −7.06, [−35.46, 21.35]	1.000, −6.96, [−59.71, 45.79]
Bilirubin	8.72 (1.01, 20.28)	0.008	0.304	0.025, 9.74, [1.02, 18.46]	0.099, 0.92, [−0.13, 1.97]	0.022, 10.66, [1.35, 19.96]
Total protein	29.08 (1.34, 29.53)	<0.001	0.569	<0.001, −9.10, [−12.91, −5.30]	1.000, −0.03, [−2.11, 2.06]	<0.001, −9.13, [−13.56, −4.71]
Albumin	26.15 (1.59, 34.94)	<0.001	0.543	<0.001, −4.99, [−7.64, −2.34]	0.109, −1.47, [−3.18, 0.24]	<0.001, −6.46, [−9.24, −3.68]
Creatinine	1.47 (1.01, 22.12)	0.238	0.063	0.723, 45.39, [−52.19, 142.98]	1.000, 2.61, [−4.70, 9.91]	0.705, 48.0, [−53.83, 149.83]
Urea	5.35 (1.02, 22.42)	0.030	0.196	0.083, 7.91, [−0.79, 16.62]	1.000, −0.20, [−1.19, 0.78]	0.097, 7.71, [−1.04, 16.46]
1,2-o-dilauryl-rac-glycero-3-glutaric acid-(6′-methylresorufin) ester lipase	5.17 (1.06, 22.29)	0.031	0.197	0.093, 158.86, [−19.80, 337.51]	1.000, −4.29, [−40.16, 31.58]	0.100, 154.56, [−22.01, 331.14]
Iron	0.07 (1.07, 22.4)	0.816	0.003	1.000, −1.582, [−25.27, 22.11]	1.000, −1.14, [−6.12, 3.84]	1.000, −2.72, [−26.51, 21.06]
C-reactive protein	39.59 (1.01, 22.12)	<0.001	0.643	<0.001, 128.34, [75.50, 181.18]	0.193, 2.48, [−0.82, 5.78]	<0.001, 130.82, [77.02, 184.62]
**Coagulation**						
Prothrombin time	2.36 (1.91, 34.35)	0.112	0.116	0.093, 0.39, [−0.05, 0.83]	1.000, −0.12, [−0.65, 0.42]	0.455, 0.274, [−0.21, 0.76]
Partial thromboplastin time	0.811 (1.31, 23.52)	0.408	0.043	0.723, 0.80, [−0.94, 2.54]	1.000, −0.23, [−1.24, 0.78]	1.000, 0.57, [−1.60, 2.74]
Thrombin time	7.45 (1.59, 28.56)	0.004	0.293	<0.001, −1.89, [−2.98, −0.80]	1.000, −0.04, [−1.82, 1.74]	0.013, −1.932, [−3.50; −0.36]
Fibrinogen	46.22 (1.18, 21.20)	<0.001	0.720	<0.001, 257.32, [159.18, 355.45]	0.016, 49.05, [8.30, 89.81]	<0.001, 306.37, [191.52, 421.22]
***Babesia* spp. antibody levels**						
Antibody levels	32.04 (1.21, 26.56)	<0.001	0.593	<0.001, −45.64, [−64.50, −26.77]	0.002, 10.71, [3.87, 17.56]	<0.001, −34.92, [−52.63, −17.22]

*Abbreviations*: CI, confidence interval; *df*, degrees of freedom; *ᶯ*^2^, partial eta squared (proportion of variance in the dependent variable explained by an effect, while removing the variance accounted for by other factors and subjects); *M*_diff_, mean difference (raw, unstandardized difference between two time points or conditions).

The dynamic analysis to address the early treatment response and the correlation analysis is demonstrated in Table 6. High positive correlations were detected for fibrinogen and CRP concentrations. Moderate positive correlations were recorded for the level of parasitemia and hematocrit. Moderate negative correlations were detected for white blood cells and CRP concentrations, for platelets and CRP concentrations, for antibody levels and bilirubin concentrations, and for antibody levels and imidocarb dipropionate dosage (Table 6).

**Table 6:** Correlation analysis (Spearman’s ρ) showing moderate (> 0.300–0.499) to strong (≥ 0.500) correlations of the dynamic analysis in selected key laboratory parameters at the time of diagnosis (T0) and the time of the second imidocarb dipropionate injection (T1) in 23 dogs with acute *Babesia canis* infections.

		Hematocrit	White blood cells	Platelets	C-reactive protein	Fibrinogen	Bilirubin	*Babesia* spp. antibody levels	Pathogen quantification
White blood cells	ρ	−0.045							
	*P*	0.840							
	*n*	23							
Platelets	ρ	0.297	0.172						
	*P*	0.168	0.433						
	*n*	23	23						
C-reactive protein	ρ	0.048	**−0.477**	**−0.448**					
	*P*	0.828	**0.022∗**	**0.032∗**					
	*n*	23	23	23					
Fibrinogen	ρ	−0.122	−0.418	−0.430	**0.832**				
	*P*	0.618	0.075	0.066	**<0.001∗∗∗**				
	*n*	19	19	19	19				
Bilirubin	ρ	0.183	−0.257	−0.234	0.318	0.436			
	*P*	0.404	0.237	0.282	0.140	0.062			
	*n*	23	23	23	23	19			
*Babesia* spp. antibody levels	ρ	0.328	0.091	0.216	−0.107	−0.008	**−0.421**		
	*P*	0.126	0.681	0.322	0.627	0.974	**0.045∗**		
	*n*	23	23	23	23	19	23		
Pathogen quantification	ρ	**0.428**	−0.081	−0.099	0.093	−0.065	0.177	0.218	
	*P*	**0.041∗**	0.715	0.654	0.673	0.792	0.418	0.318	
	*n*	23	23	23	23	19	23	23	
Dosage of imidocarb dipropionate	ρ	−0.203	−0.265	−0.240	0.260	0.157	0.019	**−0.441**	−0.099
	*P*	0.352	0.222	0.271	0.231	0.520	0.932	**0.035∗**	0.654
	*n*	23	23	23	23	19	23	23	23

*Abbreviations*: ρ, Spearman’s correlation coefficient; *P*, significance value (two-sided); *n*, number of dogs.

*Note*: Statistically significant associations are highlighted in bold with *P*-values indicated with asterisks (∗*P* < 0.05; ∗∗∗*P* < 0.001).

### 3.6 Treatment and outcome

All 23 dogs were treated with subcutaneous injections of imidocarb dipropionate at T0 and T1, and all dogs were tested PCR negative for *B. canis* at T1 and T2, respectively. The dosage was known for all 23 dogs and ranged from 1.7 mg/kg BW to 7.0 mg/kg BW (median: 4.2 mg/kg BW; SD: 1.8 mg/kg BW). The information about complicated and uncomplicated course of disease from the questionnaire was available in 16 of the 23 dogs (70%), with uncomplicated course of disease in 15 of the 16 cases (94%). One out of 16 dogs (6%) had a complicated course and required supportive care (intensive care and blood transfusions). Each dog survived the acute infection, and all 23 dogs were still alive by T2, making the mortality rate in our study 0%.

## 4 Discussion

To the best of our knowledge, our study is the first to assess clinical signs, hematological parameters, biochemistry, coagulation profiles, acute phase proteins, and *Babesia* spp. antibody levels for more than 15 days in dogs with acute *B*. *canis* infections after treatment with imidocarb dipropionate in a cohort of 23 dogs. Especially platelet counts, hematocrit, fibrinogen concentrations, and CRP concentrations differed between the different time-points of the study, representative for more than 60% of the study population, respectively. This underlines the fact that most dogs with acute *B. canis* infections are presented with an acute phase reaction with thrombocytopenia as the most common hematological abnormality.

Despite the wide range of imidocarb dipropionate dosages (1.7 mg/kg BW to 7.0 mg/kg BW), all *B. canis* PCRs were negative by T1, and hematological as well as biochemistry abnormalities significantly improved in all dogs. Currently, the FDA’s recommendation for the therapy of an acute *B. canis* infection is the administration of two injections 6.6 mg/kg BW subcutaneously or intramuscularly fortnightly (FDA, 2026). However, given all *B. canis* PCRs were already negative after the first injection in the present study, an alternative therapeutic approach with only one injection of imidocarb at 6.6 mg/kg BW should be considered. The second imidocarb dipropionate injection significantly decreased the protective *Babesia* spp. antibody levels, which may protect dogs from a severe and life-threatening course of disease during a later *B. canis* infection (Von Hohnhorst et al., 2025b). This is additionally underlined by the correlation analysis in our study. It is important to highlight the use of high-dosed imidocarb dipropionate, as the manufacturer still recommends a therapeutic dosage of 0.25 ml/10 kg BW (i.e. 3.0 mg/kg BW) and a prophylactic dosage of 0.5 ml/10 kg BW (i.e. 6.0 mg/kg BW) in the package insert in central Europe. A recent case report points out treatment failure by using lower-ranged dosages of imidocarb dipropionate in a dog with an acute *B. canis* infection in Germany, besides the use of glucocorticoids (Schäfer et al., 2026). The knowledge regarding the potential transmission of *Babesia* spp. from dogs after natural infection and treatment with imidocarb dipropionate while being PCR negative is unclear yet. However, none of *Rhipicephalus bursa* ticks feeding on treated donors in the case of *B. ovis* tested positive, and no tick transmitted the pathogen after feeding, mainly due to the low post-treatment parasitemia (Firat et al., 2025). Although it cannot be excluded completely, it is considered unlikely at the time-point of this study that *B. canis* might be spread from PCR-negative dogs to *D. reticulatus* ticks and may therefore contribute to the spread of the emerging disease.

The most important underlying causes for treatment failure in acute *B. canis* infections include lower-range therapeutic doses of imidocarb dipropionate, the application of glucocorticoids, co-morbidities/co-infections, and/or missing species differentiation, and therefore treating against another *Babesia* spp. not responsive to imidocarb dipropionate (Schäfer et al., 2026). Therefore, in dogs with acute *B. canis* infections defined by a positive PCR revealing *B. canis* in sequencing, dogs should be monitored 14 days after the first 6.6 mg/kg BW imidocarb dipropionate injection, including evaluation of the clinical status, PCR-testing on capillary or EDTA-blood, and a hematological as well as biochemical analysis. In case of a negative *B. canis* PCR and clinicopathological improvement, a second injection can be omitted and protective *Babesia* spp. antibody levels may persist for a longer period. If a dog stays PCR-positive for *B. canis* by day 14, a second injection should be considered even in a clinically healthy dog without any clinicopathological abnormalities, primarily from an epidemiological perspective. *Babesia canis* PCR-positive dogs could act as a pathogen reservoir and contribute to the spread of the pathogen (Glaser and Gothe, 1998).

Clinical signs such as lethargy and hyporexia/inappetence were in accordance with previous studies and considered typical for acute *B. canis* infections in dogs in Germany (Seibert et al., 2022; Weingart et al., 2023). However, fever was only recorded in 65% of the 23 dogs included, meaning that an acute *B. canis* infection should not be ruled out in dogs without fever. Darkened/bloody urine was only reported in 21% of 23 dogs in the present study and is most often linked to hemoglobinuria in dogs with acute *B. canis* infections. Interestingly, two dogs did not show any clinical signs but thrombocytopenia on laboratory testing. The identification of these dogs potentially acting as reservoirs may be crucial to prevent further spread of *B. canis* infections. Therefore, dogs with thrombocytopenia should be checked for *B. canis* by PCR even if no clinical signs are present.

The hematological and biochemical abnormalities detected in the present study were generally in line with previous studies. It should be highlighted that thrombocytopenia, which was most often marked, was the main hematological abnormality in all 23 dogs at T0. In case of thrombocytopenia, *B*. *canis* infections must be included in the list of differential diagnoses. Hemolytic anemia was in the past described as the most important hematological abnormality suspicious for acute *B. canis* infections, but meanwhile, *B. canis* infections should additionally be considered as a potential differential diagnosis in each dog presented with thrombocytopenia. The dogs in the present study most often showed mild anemia at T0 and T1, accompanied by mild reticulocytosis in 12 of the 23 dogs at T1. The regenerative response to anemia was classified as inappropriate due to dyserythropoiesis in the bone marrow of dogs with *B. rossi* infections (Seejarim et al., 2023; Bumby et al., 2024) and in humans with falciparum malaria (Wickramasinghe and Abdalla, 2000; Casals-Pascual et al., 2006; Joice et al., 2014). Direct bone marrow suppression by the pathogen and/or suppression of the hosts innate response are suspected causes for the inadequate regenerative responses in *B. rossi* infections (Seejarim et al., 2023). In the dogs infected with *B. canis* in our study, anemias were most often classified as mild, which may explain the mild reticulocyte responses. Anemia of inflammatory disease must be additionally considered, as dogs with *B. canis* infections are presented with most often marked CRP elevations in our study as well as in previous studies (Weingart et al., 2023; Von Hohnhorst et al., 2025b). Therefore, the pathophysiology of anemia and regenerative response in acute *B. canis* infections is most likely multifactorial. The classic picture of acute *B. canis* infections, consisting of pale mucous membranes and a severe anemia seems to have shifted towards a severe thrombocytopenia with concurrent mild anemia. Thrombocytes are the first cell population to drop significantly at two days post-infection (PI) while changes in erythrocytes and leukocytes are usually noted a day later (Schetters et al., 2009). This, in combination with earlier presentation to a veterinarian, could be an explanation for the laboratory shift described above. Individual immunological response and infection with different *B. canis* genotypes could impact the clinicopathological abnormalities seen in acute *B. canis* infections (Carcy et al., 2015; Helm et al., 2022; Von Hohnhorst et al., 2025b).

Interestingly, differences in concentrations of all individual blood cell types between T0 and T1 were significant, while no significant impact on blood parameters was noted comparing T1 and T2. This indicates that the recovery of the laboratory parameters is mainly achieved in the timeframe after the first imidocarb dipropionate injection. Thrombocytopenia and leukopenia both normalized more quickly than anemia, which is in line with other studies (Máthé et al., 2006a; Matijatko et al., 2007).

Twenty-five percent of our study population had a positive Coombs’ test at T0. Due to the lack of microscopic evaluation of erythrocytes/spherocytes, a definitive IMHA diagnosis could not be carried out according to the consensus guidelines (Garden et al., 2019). However, anemia paired with a positive Coombs’ test and hyperbilirubinemia is highly suggestive of an IMHA (Garden et al., 2019). The use of immunosuppressants (i.e prednisolone) in cases of acute babesiosis is controversial (Ayoob et al., 2010; Solano-Gallego et al., 2016; Nevidnyk-Pravda and Ushakova, 2025) and may lead to an increase in the *B. canis* parasite load as well as treatment failure (Schäfer et al., 2026). A strong negative correlation was detected between hematocrit and bilirubin, indicating hemolytic anemia as the most likely cause. Additionally, the level of parasitemia showed strong positive correlation with the bilirubin concentrations.

The most common biochemical abnormalities lined up with previous studies and consisted of hyperbilirubinemia, increased liver enzymes and CK elevations. Additionally, dogs showed azotemia, decreased iron and total protein values, and increased DGGR-lipase activities (Máthé et al., 2006b; Seibert et al., 2022; Weingart et al., 2023). Hyperbilirubinemia was most probably prehepatic because of hemolysis, as it affected almost only dogs with concurrent anemia (Seibert et al., 2022). Prerenal causes seem the most probable in our azotemic dogs, also referring to the urea-to-creatinine-ratios as 39% of dogs showed prolonged capillary refill times, mainly through shock or gastrointestinal losses in acute *B. canis* infections (Medaille et al., 2004).

Hypoalbuminemia was not frequent, but albumin may be interpreted as a negative acute-phase protein (Solano-Gallego et al., 2008; Cray et al., 2009; Von Hohnhorst et al., 2025b), fitting well with the most often marked acute-phase response in the majority of dogs in the present study due to an increase in CRP. Increased DGGR-lipase activities can be indicative of pancreatitis, and as it increases mortality in *B. rossi* infections, it should also be monitored and addressed (Möhr et al., 2000). Just as for the hematological abnormalities discussed previously, all biochemical parameters mentioned above showed a quick and significant recovery from T0 to T1, but no further significance was demonstrated comparing T1 and T2.

As expected, most dogs (87%) showed a systemic inflammation reflected by the initial CRP elevations in the present study. The CRP, as a major acute-phase protein, is a marker for the severity of an inflammation and might be helpful for monitoring the response to treatment with imidocarb dipropionate (Matijatko et al., 2007; Cray et al., 2009). Fibrinogen, initially increased in most of our dogs, is another indicator of systemic inflammation in acute babesiosis (Ruiz de Gopegui et al., 2007; Torrente et al., 2015). Both parameters showed a quick recovery after treatment and had normalized in almost all dogs between T0 and T1 (with no significant differences between T1 and T2). Recent research has shown that, besides CRP, fibrinogen and albumin, iron can be an indicator of systemic inflammation in dogs and a tool for monitoring inflammation (Torrente et al., 2015). Hypoferremia occurs in response to inflammatory cytokines mediating the production of hepcidin, which then inhibits the export of iron from cells to the plasma (Torrente et al., 2015). Most dogs initially showed hypoferremia in the present study, which subsided quickly after treatment. After dietary uptake, iron is constantly stored and reused within the body. As dogs with acute babesiosis do not usually experience a veritable loss of iron (e.g. through chronic external haemorrhage) and hypoferremia is most often linked to the inflammation (thus showing quick recovery) in these cases, iron substitution is not routinely advised in dogs with acute *B. canis* infections.

CRP concentrations negatively correlated with *Babesia* spp. antibody levels, indicating that dogs with higher *Babesia* spp. antibody levels had less severe CRP elevations (Von Hohnhorst et al., 2025b). The dogs that tested serologically positive in that study were most often imported individuals. This confirms the presence of protective antibodies in predominantly imported dogs in Germany. The present findings corroborated these results, as all dogs with positive *Babesia* antibody levels were imported to Germany. Dogs with positive antibody levels showed less severe clinical signs and showed only mild laboratory abnormalities. On the other hand, almost all serologically negative dogs at T0 had not been imported, indicating that the canine population in Germany is predominantly immunologically naïve (Von Hohnhorst et al., 2025b). Most of these initially serologically negative dogs formed positive *B. canis* antibody levels by T1 in the present study. Interestingly, these antibody levels decreased again in most of the study cohort by T2. One explanation for the antibody levels dropping could be the second injection of imidocarb dipropionate. As the antibodies are believed to be of a protective nature and a decrease could result in a weaker immune response in case of re-infection, a novel therapeutic approach with the administration of one dose of imidocarb should be considered, as described above.

Finally, coagulation pathologies like DIC and/or SIRS are described as life-threatening complications in acute *B. canis* infections. In the present study, none of the 23 dogs showed a prolonged PT, while prolonged PTT was rarely identified. Hypofibrinogenemia occurred in one dog, while most showed hyperfibrinogenemia. It cannot be ruled out that fibrinogen synthesis due to the concurrent inflammation outweighed fibrinogen consumption while DIC was occurring, but it seems highly unlikely due to the other physiological parameter values. Previous studies on this topic mainly used data from referral clinics, and since our study population consisted of dogs diagnosed in practices with mainly uncomplicated courses, discrepancies might arise.

Limitations in the present study are mainly linked to the relatively small number of dogs included, which might impact the representativeness of the population. Therefore, it is advisable to confirm the results of the present study in other studies with more dogs included. Clinical data were available in all 23 dogs at T0 but only in 5 of the 23 dogs (22%) at T1 and in 6 of the 23 dogs (26%) at T2. However, information on the course of disease was available in 16 of the 23 dogs (70%) from the questionnaires. The *B. canis* strains detected in the present study were not further subclassified, and different strains may have a significant impact on the severity of clinicopathological abnormalities in dogs with acute *B. canis* infections. Co-infections potentially impacting clinicopathological abnormalities, the course of disease, and the outcome were ruled out as best as possible in all 23 dogs.

## 5 Conclusions

Acute *B. canis* infections should not be ruled out in dogs without fever, as well as without marked hemolytic anemia and hemoglobinuria. Thrombocytopenia, which was most often marked, was the most important hematological abnormality in dogs with acute *B. canis* infections in the present study. In general, dogs with thrombocytopenia should be checked for *B. canis* infections by PCR on EDTA and/or capillary blood. In dogs with high *Babesia* antibody levels, mild thrombocytopenia, and no clinical signs, PCR testing for *B. canis* is highly recommended to prevent further spread of the disease by these asymptomatic carriers. Dogs with acute *B. canis* infections should be treated with 6.6 mg/kg BW imidocarb dipropionate as recommended by the FDA. Besides the wide range of dosage in the present study, all laboratory abnormalities showed a quick and significant recovery and most initially serologically negative dogs developed positive *Babesia* antibody levels in the first two weeks after diagnosis and start of treatment with imidocarb dipropionate. Laboratory abnormalities did not change significantly after the second imidocarb injection, but *Babesia* antibody levels decreased, most likely due to the second imidocarb dipropionate injection, leading to less protection against severe disease in dogs in case of future *B. canis* infections. This is supported by the fact that imported dogs with high antibody levels showed less severe clinicopathological abnormalities than dogs without import anamnesis, which are still predominantly seronegative in Germany. Therefore, it is recommended to monitor dogs with acute *B. canis* infections two weeks after the first imidocarb dipropionate injection by evaluation of the clinical status, hematology, biochemistry, including CRP, and PCR testing for *B. canis*. If clinicopathological abnormalities resolve or significantly improve and PCR testing is negative, no second imidocarb dipropionate injection might be recommended, as no significant improvement of clinicopathological abnormalities was demonstrated by a second injection in the present study.

## Ethical approval

Not applicable.

## CRediT authorship contribution statement

**Clara M. Eisenecker:** Investigation, Visualization, Writing - original draft. **Andreas Moritz:** Supervision, Writing - review & editing. **Imke M. von Hohnhorst:** Writing - review & editing. **Christina Strube:** Writing - review & editing. **Elisabeth Müller:** Writing - review & editing. **Ingo Schäfer:** Conceptualization, Supervision, Writing - review & editing.

## Funding

This research did not receive any specific grant from funding agencies in the public, commercial, or not-for-profit sectors.

## Declaration of competing interests

The authors declare the following financial interests/personal relationships which may be considered as potential competing interests: Ingo Schäfer is an employee, and Elisabeth Müller is the CEO of Laboklin GmbH & Co. KG; Andreas Moritz, Christina Strube, and Ingo Schäfer have repeatedly lectured for and/or acted as consultants for diagnostic and (veterinary) pharmaceutical companies; Andreas Moritz and Christina Strube have previous and ongoing research collaborations with various diagnostic and (veterinary) pharmaceutical companies. The other authors declare that they have no known competing financial interests or personal relationships that could have appeared to influence the work reported in this paper.

## Data Availability

All data generated or analyzed during this study are included in this published article and its supplementary files.

## References

[bib1] Ayoob A.L., Hackner S.G., Prittie J. (2010). Clinical management of canine babesiosis. J. Vet. Emerg. Crit. Care. (San Antonio).

[bib2] Bajer A., Beck A., Beck R., Behnke J.M., Dwuznik-Szarek D., Eichenberger R.M. (2022). Babesiosis in southeastern, central and northeastern Europe: an emerging and re-emerging tick-borne disease of humans and animals. Microorganisms.

[bib3] Beletic A., Janjic F., Radakovic M., Spariosu K., Francuski Andric J. (2021). Systemic inflammatory response syndrome in dogs naturally infected with *Babesia canis*: association with the parasite load and host factors. Vet. Parasitol..

[bib4] Birkenheuer A.J., Levy M.G., Stebbins M., Poore M., Breitschwerdt E. (2003). Serosurvey of anti*Babesia* antibodies in stray dogs and American pit bull terriers and American staffordshire terriers from North Carolina. J. Am. Anim. Hosp. Assoc..

[bib5] Bumby M.M., Clift S.J., Hooijberg E.H., Leisewitz A.L. (2024). Cytological and histopathological bone marrow findings in dogs with natural *Babesia rossi* infection. J. S. Afr. Vet. Assoc..

[bib6] Camacho A.T., Guitian E.J., Pallas E., Gestal J.J., Olmeda A.S., Goethert H.K. (2004). Azotemia and mortality among *Babesia microti*-like infected dogs. J. Vet. Intern. Med..

[bib7] Carcy B., Randazzo S., Depoix D., Adaszek L., Cardoso L., Baneth G. (2015). Classification of *Babesia canis* strains in Europe based on polymorphism of the *Bc28.1*-gene from the *Babesia canis Bc28* multigene family. Vet. Parasitol..

[bib8] Casals-Pascual C., Kai O., Cheung J.O., Williams S., Lowe B., Nyanoti M. (2006). Suppression of erythropoiesis in malarial anemia is associated with hemozoin *in vitro* and *in vivo*. Blood.

[bib9] Cohen J. (1988).

[bib10] Cray C., Zaias J., Altman N.H. (2009). Acute phase response in animals: a review. Comp. Med..

[bib11] Diakou A. (2024). Biting back: advances in fighting ticks and understanding tick-borne pathogens. Pathogens.

[bib12] Dubova O.A., Feshchenko D.V., Yevstafieva V.O., Melnychuk V.V., Dubovyi A.A. (2023). Pathogenic relationship between kidney pathologies and the microcirculatory capillary layer in dogs under the influence of *Babesia canis*. Regul. Mech. Biosyst..

[bib13] FDA (2026). https://animaldrugsatfda.fda.gov/adafda/views/#/home/previewsearch/141-071.

[bib14] Firat R., Ulucesme M.C., Eyvaz A., Alatas M., Aktas M., Ceylan O. (2025). Persistence and transmission dynamics of *Babesia ovi*s after imidocarb dipropionate treatment: evaluation *via* blood transfusion and tick infestation. Pathogens.

[bib15] Garden O.A., Kidd L., Mexas A.M., Chang Y.M., Jeffery U., Blois S.L. (2019). ACVIM consensus statement on the diagnosis of immune-mediated hemolytic anemia in dogs and cats. J. Vet. Intern. Med..

[bib16] Glaser B., Gothe R. (1998). [Imported arthropod-borne parasites and parasitic arthropods in dogs. Species spectrum and epidemiologic analysis of the cases diagnosed in 1995/96]. Tierarztl. Prax. K. Kleintiere Heimtiere.

[bib17] Gray J.S., Dautel H., Estrada-Pena A., Kahl O., Lindgren E. (2009). Effects of climate change on ticks and tick-borne diseases in Europe. Interdiscip. Perspect. Infect. Dis..

[bib18] Helm C., Weingart C., Ramünke S., Schäfer I., Müller E., Samson-Himmelstjerna G.V. (2022). High genetic diversity of *Babesia canis* (Piana & Galli-Valerio, 1895) in a recent local outbreak in Berlin/Brandenburg, Germany. Transbound. Emerg. Dis..

[bib19] Jefferies R., Ryan U.M., Muhlnickel C.J., Irwin P.J. (2003). Two species of canine *Babesia* in Australia: detection and characterization by PCR. J. Parasitol..

[bib20] Joice R., Nilsson S.K., Montgomery J., Dankwa S., Egan E., Morahan B. (2014). *Plasmodium falciparum* transmission stages accumulate in the human bone marrow. Sci. Transl. Med..

[bib21] Kivrane A., Namina A., Seleznova M., Akopjana S., Capligina V., Ranka R. (2021). Development of a real-time PCR method for rapid diagnosis of canine babesiosis and anaplasmosis. Parasites Vectors.

[bib22] Leisewitz A.L., Mrljak V., Dear J.D., Birkenheuer A. (2023). The diverse pathogenicity of various *Babesia* parasite species that infect dogs. Pathogens.

[bib23] Maggi R.G., Birkenheuer A.J., Hegarty B.C., Bradley J.M., Levy M.G., Breitschwerdt E.B. (2014). Comparison of serological and molecular panels for diagnosis of vector-borne diseases in dogs. Parasites Vectors.

[bib26] Matijatko V., Kis I., Torti M., Brkljacic M., Kucer N., Rafaj R.B. (2009). Septic shock in canine babesiosis. Vet. Parasitol..

[bib27] Matijatko V., Kis I., Torti M., Brkljacic M., Rafaj R.B., Zvorc Z., Mrljak V. (2010). Systematic inflammatory response syndrome and multiple organ dysfunction syndrome in canine babesiosis. Vet. Arhiv.

[bib28] Matijatko V., Mrljak V., Kis I., Kucer N., Forsek J., Zivicnjak T. (2007). Evidence of an acute phase response in dogs naturally infected with *Babesia canis*. Vet. Parasitol..

[bib29] Medaille C., Trumel C., Concordet D., Vergez F., Braun J.P. (2004). Comparison of plasma/serum urea and creatinine concentrations in the dog: a 5-year retrospective study in a commercial veterinary clinical pathology laboratory. J. Vet. Med. A Physiol. Pathol. Clin. Med..

[bib31] Möhr A.J., Lobetti R.G., van der Lugt J.J. (2000). Acute pancreatitis: a newly recognised potential complication of canine babesiosis. J. S. Afr. Vet. Assoc..

[bib30] Mierzejewska E.J., Estrada-Peña A., Bajer A. (2017). Spread of *Dermacentor reticulatus* is associated with the loss of forest area. Exp. Appl. Acarol..

[bib32] Moubri K., Kleuskens J., Van de Crommert J., Scholtes N., Van Kasteren T., Delbecq S. (2018). Discovery of a recombinant *Babesia canis* supernatant antigen that protects dogs against virulent challenge infection. Vet. Parasitol..

[bib25] Máthé A., Vörös K., Papp L., Reiczigel J. (2006). Clinical manifestations of canine babesiosis in Hungary (63 cases). Acta Vet. Hung..

[bib24] Máthé Á., Vörös K., Németh T., Biksi I., Hetyey C., Manczur F., Tekes L. (2006). Clinicopathological changes and effect of imidocarb therapy in dogs experimentally infected with *Babesia canis*. Acta Vet. Hung..

[bib33] Nevidnyk-Pravda A.Y., Ushakova H.O. (2025). Effect of combined therapy with imidocarb and prednisolone on hematological parameters in dogs infected with *Babesia canis*. JVMBBS.

[bib34] Olmeda A.S., Armstrong P.M., Rosenthal B.M., Valladares B., del Castillo A., Armas F.d. (1997). A subtropical case of human babesiosis. Acta Trop..

[bib35] Ruiz de Gopegui R., Penalba B., Goicoa A., Espada Y., Fidalgo L.E., Espino L. (2007). Clinico-pathological findings and coagulation disorders in 45 cases of canine babesiosis in Spain. Vet. J..

[bib38] Schetters T.P., Kleuskens J.A., Van De Crommert J., De Leeuw P.W., Finizio A.L., Gorenflot A. (2009). Systemic inflammatory responses in dogs experimentally infected with *Babesia canis*; a haematological study. Vet. Parasitol..

[bib36] Schäfer I., Baitis V., Strube C., Jindal K., Müller E., Naucke T.J., Moritz A. (2026). Treatment failure in a dog with acute *Babesia canis* infection using lower-range therapeutic doses of imidocarb dipropionate. Parasitol. Res..

[bib37] Schäfer I., Helm C.S., Von Samson-Himmelstjerna G., Krücken J., Kottmann T., Holtdirk A. (2023). Molecular detection of *Babesia* spp. in dogs in Germany (2007–2020) and identification of potential risk factors for infection. Parasites Vectors.

[bib39] Seejarim C., Rautenbach Y., Hooijberg E.H., Leisewitz A.L., Schoeman J.P., Goddard A. (2023). Regenerative response in dogs naturally and experimentally infected with *Babesia rossi*. Vet. Clin. Pathol..

[bib40] Seibert S., Rohrberg A., Stockinger A., Schaalo S., Marz I. (2022). [Occurrence of canine babesiosis in dogs in the Rhine-Main area of Hesse, Germany - a case study of 81 dogs]. Tierarztl. Prax. Ausg. K. Kleintiere Heimtiere.

[bib41] Solano-Gallego L., Sainz A., Roura X., Estrada-Pena A., Miro G. (2016). A review of canine babesiosis: the European perspective. Parasites Vectors.

[bib42] Solano-Gallego L., Trotta M., Carli E., Carcy B., Caldin M., Furlanello T. (2008). *Babesia canis canis* and *Babesia canis vogeli* clinicopathological findings and DNA detection by means of PCR-RFLP in blood from Italian dogs suspected of tick-borne disease. Vet. Parasitol..

[bib43] Spariosu K., Janjic F., Andric J.F., Radakovic M., Beletic A., Kovacevic Filipovic M., Milanovic S. (2021). Relationship between changes in hematological parameters, levels of acute phase proteins and redox homeostasis during acute *Babesia canis* infection in dogs. Acta Vet. (Beograd).

[bib44] Torrente C., Manzanilla E.G., Bosch L., Fresno L., Rivera Del Alamo M., Andaluz A. (2015). Plasma iron, C-reactive protein, albumin, and plasma fibrinogen concentrations in dogs with systemic inflammatory response syndrome. J. Vet. Emerg. Crit. Care (San Antonio).

[bib45] Von Hohnhorst I.M., Moritz A., Eisenecker C.M., Strube C., Naucke T.J., Müller E., Schäfer I. (2025). Apolipoprotein A-1 does not appear to be a suitable acute-phase reaction marker in canine babesiosis and hemoplasmosis. Curr. Res. Parasitol. Vector Borne Dis..

[bib46] Von Hohnhorst I.M., Moritz A., Eisenecker C.M., Strube C., Rodjana K., Müller E., Schäfer I. (2025). Impact of pathogen quantification, antibody levels, and stays abroad on hematological as well as biochemistry parameters and acute-phase proteins in dogs with acute *Babesia canis* infections in Germany. Parasites Vectors.

[bib47] Weingart C., Helm C.S., Müller E., Schäfer I., Skrodzki M., von Samson-Himmelstjerna G. (2023). Autochthonous *Babesia canis* infections in 49 dogs in Germany. J. Vet. Intern. Med..

[bib48] Wickramasinghe S.N., Abdalla S.H. (2000). Blood and bone marrow changes in malaria. Baillieres Best Pract. Res. Clin. Haematol..

