# Supplementary material for: Clinicopathological abnormalities and outcome of acute Babesia canis infections in 23 dogs treated with imidocarb dipropionate
Source: Curr Res Parasitol Vector Borne Dis. 2026 Jun 2;10:100395. doi: 10.1016/j.crpvbd.2026.100395 (PMC13311271; doi:10.1016/j.crpvbd.2026.100395)
Supplement: Multimedia component 1 [file mmc1.pdf]

Supplementary Table S1. Laboratory parameters with statistically significant differences between the three timepoints T0, T1, and T2, in 23 dogs with acute *Babesia canis* infections (decreased parameters according to reference intervals: n/N (%); increased parameters according to reference intervals: n/N (%); median; standard deviation; interquartile range; minimum-maximum).

**Parameter (reference interval)**[a]	**T0 (first diagnosis and first imidocarb dipropionate injection)**	**T1 (second imidocarb dipropionate injection, median time to T0 15 days)**	**T2 (checkup, median time to T0 32 days)**	**Statistically significant differences ($P < 0.05$)**[b]
**Hematology**[c]				
Hematocrit (0.44–0.52 L/L)	19/23 (83); 2/23 (9); 0.34; 0.10; 0.11; 0.19–0.58	12/23 (52); 0/23 (0); 0.43; 0.10; 0.11; 0.35–0.56	3/23 (13); 2/23 (9); 0.47; 0.10; 0.05; 0.37–0.55	T0 *vs* T1: $P = 0.003$<br>T0 *vs* T2: $P < 0.001$
Reticulocytes (< 110 µL)	0/23 (0); 0/23 (0); 27.3; 22.7; 30.7; 9.0–98.0	0/23 (0); 12/23 (52); 139.4; 111.3; 152.1; 18.0–399.0	0/23 (0); 3/23 (13); 76; 30.6; 32.1; 27.0–159.0	T0 *vs* T1: $P < 0.001$<br>T0 *vs* T2: $P < 0.001$
White blood cells (6.0–12.0 G/L)	13/23 (57); 1/23 (4); 5.5; 3.3; 5.3; 1.1–13.0	5/23 (22); 5/23 (22); 9.7; 3.7; 5.7; 4.4–18.1	0/23 (0); 4/23 (17); 9.5; 2.2; 2.0; 5.3–13.3	T0 *vs* T1: $P = 0.010$<br>T0 *vs* T2: $P < 0.001$
Segmented neutrophilic granulocytes (3.0–11.5 G/L)	10/23 (43); 0/23 (0); 3.1; 1.9; 2.9; 0.9–8.6	1/23 (4); 1/23 (4); 5.4; 2.9; 3.6; 2.8–13.5	1/23 (4); 0/23 (0); 5.3; 1.4; 1.7; 2.9–8.6	T0 *vs* T1: $P < 0.001$<br>T0 *vs* T2: $P = 0.002$
Lymphocytes (1.0–3.6 G/L)	11/23 (48); 1/23 (4); 0.9; 1.2; 1.5; 0.1–4.7	0/23 (0); 3/23 (13); 2.4; 1.4; 1.8; 1.2–6.3	0/23 (0); 3/23 (13); 2.7; 1.1; 1.4; 1.2–5.2	T0 *vs* T1: $P = 0.001$<br>T0 *vs* T2: $P < 0.001$
Eosinophilic granulocytes (0.04–0.60 G/L)	1/23 (4); 16/23 (70); 0.2; 0.1; 0.0–0.7	2/23 (9); 10/23 (43); 0.6; 0.2; 0.6; 0.0–1.3	1/23 (4); 13/23 (43); 0.7; 0.5; 0.4; 0.1–2.3	T0 *vs* T1: $P < 0.001$<br>T0 *vs* T2: $P < 0.001$
Thrombocytes (150–500 G/L)	23/23 (100); 0/23 (0); 33; 38.1; 39; 11–149	2/23 (9); 1/23 (4); 289; 124.3; 191; 85–547	3/23 (13); 0/23 (0); 268; 75.7; 128; 110–403	T0 *vs* T1: $P < 0.001$<br>T0 *vs* T2: $P < 0.001$
**Biochemistry**[d]				
Alkaline phosphatase (< 147 U/L)	0/23 (0);12/23 (52); 149; 64.8; 111.0; 18–267	0/23 (0); 4/23 (17); 90; 166.2; 59.8; 19–865	0/23 (0); 3/23 (13); 58; 125.2; 81; 21–624	T0 *vs* T2: $P = 0.003$
Aspartate aminotransferase (< 51 U/L)	0/23 (0); 17/23 (74); 76.8; 82.9; 154.2; 17–292	0/22 (0); 0/22 (0); 25.3; 25.7; 4.9; 14–38	0/23 (0); 1/23 (4); 25.7; 10.0; 7.8; 18–54	T0 *vs* T1: $P < 0.001$<br>T0 *vs* T2: $P < 0.001$
Creatine kinase (< 200 U/L)	0/23 (0); 14/23 (61); 237; 1049.8; 391.0; 41–5214	0/23 (0); 0/23 (0); 88; 39.2; 64.0; 46–188	0/23 (0); 4/23 (17); 90; 71.0; 43; 50–263	T0 *vs* T1: $P < 0.001$<br>T0 *vs* T2: $P = 0.001$
Bilirubin (< 3.4 µmol/L)	0/23 (0); 18/23 (78); 6.2; 18.6; 10.2; 0.6–77.9	0/22 (0); 1/22 (5); 1.3; 2.0; 1.0; 0.1–7.6	0/22 (0); 0/22 (0); 1.1; 0.5; 0.6; 0.3–1.9	T0 *vs* T1: $P < 0.001$<br>T0 *vs* T2: $P < 0.001$
Total protein (54–75 g/L)	11/23 (48); 0/23 (0); 54.2; 6.9; 13.0; 43–67	0/23 (0); 0/23 (0); 61.3; 4.9; 9.1; 55–71	1/23 (4); 0/23 (0); 62.0; 4.9; 6.0; 49–70	T0 *vs* T1: $P < 0.001$<br>T0 *vs* T2: $P <0.001$
Albumin (25–44 g/L)	2/23 (9); 0/23 (0); 29.3; 5.3; 6.9; 22–43	0/23 (0); 0/23 (0); 35.9; 3.9; 2.9; 25.8–44.0	0/23 (0); 0/23 (0); 37.3; 2.7; 3.0; 29.4–42.6	T0 *vs* T1: $P = 0.007$<br>T0 *vs* T2: $P < 0.001$
1,2-o-dilauryl-rac-glycero-3-glutaric acid-(6′-methylresorufin) ester lipase (< 120 U/L)	0/23 (0); 9/23 (39); 80.0; 315.7; 235.0; 14.0–1093.0	0/22 (0); 3/22 (14); 38.9; 49.1; 42.2; 2.0–235.0	0/23 (0); 1/23 (4); 45.6; 47.2; 50.9; 26.0–217.0	T0 *vs* T1: $P = 0.034$

Parameter (reference interval)[a]	T0 (first diagnosis and first imidocarb dipropionate injection)	T1 (second imidocarb dipropionate injection, median time to T0 15 days)	T2 (checkup, median time to T0 32 days)	Statistically significant differences ($P < 0.05$)[b]
Calcium (2.3–3.0 mmol/L)	12/23 (52); 0/23 (0); 2.2; 0.3; 0.4; 1.8–3.0	1/23 (4); 1/23 (4); 2.6; 0.6; 0.2; 0.3–3.4	0/23 (0); 0/23 (0); 2.6; 0.1; 0.1; 2.4–2.8	T0 *vs* T1: $P = 0.003$<br>T0 *vs* T2: $P < 0.001$
Iron (15–45 µmol/L)	14/23 (61); 0/23 (0); 14.5; 39.5; 193.4; 6–202	2/22 (9); 3/22 (14); 22.3; 12.6; 16.8; 14–58	1/23 (4); 1/23 (4); 28.7; 8.3; 10.0; 13–46	T0 *vs* T1: $P = 0.046$<br>T0 *vs* T2: $P = 0.002$
Potassium (3.5–5.1 mmol/L)	1/23 (4); 2/23 (9); 4.2; 0.5; 0.6; 3.4–5.4	0/22 (0); 5/22 (23); 5.0; 0.4; 0.7; 4.2–5.7	0/23 (0); 5/23 (22); 4.8; 0.5; 0.5; 4.2–6.0	T0 *vs* T1: $P < 0.001$<br>T0 *vs* T2: $P < 0.001$
C-reactive protein (< 15 mg/L)[e]	0/23 (0); 20/23 (87); 108.1; 101.0; 161.4; 0.2–401	0/23 (0); 1/23 (4); 4.3; 6.6; 3.5; 0.7–32	0/23 (0); 1/23 (4); 1.8; 3.5; 3.1; 0.1–15	T0 *vs* T1: $P < 0.001$<br>T0 *vs* T2: $P < 0.001$
**Coagulation status**[f]				
Thrombin time (10.0–18.3 s)	3/19 (16); 0/19 (0); 11.4; 1.2; 4.8; 8.5–13.3	0/19 (0); 0/19 (0); 12.3; 1.5; 1.9; 10.9–17.2	0/19 (0); 1/19 (5); 12.0; 2.3; 1.7; 11.3–20.6	T0 *vs* T1: $P < 0.001$<br>T0 *vs* T2: $P = 0.002$
Fibrinogen (130–310 mg/dL)	1/19 (5); 16/19 (84); 575; 191.7; 168.0; 107–856	0/19 (0); 7/19 (37); 262; 75.9; 100.0; 138–450	1/19 (5); 1/19 (5); 236; 66.5; 77.0; 96–378	T0 *vs* T1: $P = 0.003$<br>T0 *vs* T2: $P < 0.001$
***Babesia* spp. antibody levels**[g]				
Antibody level (< 19.0 technical units)	Negative 20/23 (87); positive 3/23 (13); 2.4; 33.2; 10.3; 0.1–115.8	Negative 1/23 (4); positive 22/23 (96); 64.9; 28.8; 40.6; 5.5–121.8	Negative 2/23 (9); positive 21/23 (91); 25.1; 33.4; 0.1–110.6	T0 *vs* T1: $P < 0.001$<br>T0 *vs* T2: $P = 0.001$

[a] Reference intervals according to the Laboklin laboratory (Bad Kissingen, Germany).

[b] Kruskal-Wallis test including Bonferroni correction.

[c] Sysmex XN-V analyzer, Sysmex Deutschland, Norderstedt, Germany (on EDTA-blood).

[d] Cobas 8000, Roche Deutschland Holding GmbH, Mannheim, Germany (on serum).

[e] Gentian Canine CRP Immunoassay, gentian diagnostics, Moos, Norway (on serum).

[f] STA Compact Max3, DIAGNOSTICA STAGO, Asnieres sur Seine, France (on citrated blood).

[g] *Babesia* ELISA Dog, Afosa, Blankenfelde-Mahlow, Germany (on serum).
